# Approaches to variant discovery for conifer transcriptome sequencing

**DOI:** 10.1371/journal.pone.0205835

**Published:** 2018-11-05

**Authors:** Emily Telfer, Natalie Graham, Lucy Macdonald, Shane Sturrock, Phillip Wilcox, Lisa Stanbra

**Affiliations:** 1 New Zealand Forest Research Institute LTD. trading as Scion, Rotorua, New Zealand; 2 Real Time Genomics, Hamilton, New Zealand; 3 Department of Mathematics and Statistics, University of Otago, Dunedin, New Zealand; CNRS UMR7622 & University Paris 6 Pierre-et-Marie-Curie, FRANCE

## Abstract

There is a wide diversity of bioinformatic tools available for the assembly of next generation sequence and subsequence variant calling to identify genetic markers at scale. Integration of genomics tools such as genomic selection, association studies, pedigree analysis and analysis of genetic diversity, into operational breeding is a goal for New Zealand’s most widely planted exotic tree species, *Pinus radiata*. In the absence of full reference genomes for large megagenomes such as in conifers, RNA sequencing in a range of genotypes and tissue types, offers a rich source of genetic markers for downstream application. We compared nine different assembler and variant calling software combinations in a single transcriptomic library and found that Single Nucleotide Polymorphism (SNPs) discovery could vary by as much as an order of magnitude (8,061 SNPs up to 86,815 SNPs). The assembler with the best realignment of the packages trialled, Trinity, in combination with several variant callers was then applied to a much larger multi-genotype, multi-tissue transcriptome and identified 683,135 *in silico* SNPs across a predicted 449,951 exons when mapped to the *Pinus taeda* ver 1.01e reference.

## Introduction

Radiata pine (*Pinus radiata* D.Don) is New Zealand’s most widely planted exotic forestry species [1] and breeding programmes are moving towards the implementation of genomics technologies to deliver genetic gains through selective breeding for traits of importance. Prior to the advent of Next Generation Sequencing platforms, Expressed Sequence Tag (EST) libraries, based on captured and sequenced cDNA have been a mainstay of gene discovery and functional genomics [2, 3]. Expressed Sequence Tag (EST) libraries have long been a rich resource for markers such as microsatellites or Simple Sequence Repeats (SSRs) [4, 5]. Indeed, the conserved nature of gene sequence across conifers has meant that frequently, EST based markers from one species can be studied in related species, giving insight to evolutionary processes and increasing the pool of available markers across a genus [6–8]. Fortunately, Next Generation Sequencing (NGS) is changing the face of molecular biology and marker discovery [9–11]. At its inception in 2006, the Illumina platform generated average read lengths of 35 bases and 1 Gigabase (Gb) of sequence in a single run. The latest HiSeq and Miseq instruments and associated chemistries (Illumina, San Diego, USA) are now generating read lengths up to 300 bases and up to 1500 Gb of sequence per run [12]. Long-read third-generation technologies [13, 14] are generating even more impressive sequence lengths, albeit with diminished base-calling accuracy.

Within a single NGS experiment, it is possible to 1) generate *de novo* sequence, and 2) perform polymorphism discovery. Ideally, genomic resources are generated via whole genome sequencing (WGS) to capture variation in both genic and non-genic space, however, due to the enormous size of conifer genomes [15–17], which can exceed 30 Gb, various methods of reduced representation sequencing [18, 19] are frequently utilised to reduce costs, while still providing a genome-wide snapshot of the variation. Focusing on transcriptomic sequence allows for the generation of contiguous consensus sequences (contigs) that can be mined for polymorphic loci and provide a catalogue of gene space, even in the absence of a species-specific reference genome [20].

The goal of genomic selection is to overcome the need for long breeding cycles, minimise dependency on expensive field trials, and increase the speed of genetic gain through selective breeding. Analysis of various deployment scenarios for genomic selection in conifers suggests potential increases in delivery of genetic gain of 80% or more, driven in particular via shortening the breeding cycle [21].

For genomic selection to be a viable strategy, sufficient numbers of well-spaced DNA markers must be identified [22, 23]. Currently, the only DNA markers present at sufficient frequency, combined with ease of identification and a variety of screening platforms [24], are single nucleotide polymorphisms (SNPs) [25]. Many markers are not suitable for genotyping, either due to technical limitations of an assay platform or due to lack of relevance to the populations of interest, therefore, an extensive resource of high quality SNPs is required for the development of high density genotyping panels, integral to genomic selection approaches [26]. In eucalypts, for example, ~47 million SNP markers were identified and screened before 60,904 were committed to a multi-species SNP array [27]. Over 500,000 SNPs were vetted to produce the OvineSNP50 bead chip [28].

Genomic selection assumes that at least some of the markers will be in linkage disequilibrium (LD) with the traits of interest [26]. Therefore, the interrogation of transcriptomes for these sequence variants assumes that much of the genetic variation of interest will be in LD with expressed genes captured in the transcriptome [29–31]. To maximise the number of SNPs detected, we investigated transcriptomes from a range of tissues and genotypes. While selection of tissue types was based on those more likely to be expressing genes regulating our key traits, growth rate, wood density and needle health, ultimately good genome coverage is more important for genomic selection than the identification of specific causative quantitative trait nucleotides (QTN) [22].

Such is the power of NGS technology that the generation of large sequencing datasets has ceased to be the research bottleneck; fast and effective bioinformatic processing of the NGS datasets is now the focus of many groups. Unlike the hardware and chemistry developed to generate these datasets, much of the analysis software being developed is freely available, including a wide variety of bioinformatics tools available for sequence assembly and *in silico* polymorphism discovery [32]. Therefore we sought to determine the most appropriate method for large scale analyses in multiple datasets [33]. Conifer genomes are very large and with latest estimates of gene models in excess of 50,000 in *P*. *taeda* [34], we compared the ease of use and performance of several publicly available global and local short read sequence alignment tools. In combination with various polymorphic prediction software, we tested a single dataset from a single genotype, prior to selecting a preferred method for application within our larger multi-genotype, multi-tissue transcriptome sequencing dataset. Here we report on the generation of the first large scale SNP marker resource for radiata pine, developed using this variant calling workflow.

## Methods

### Tissue collection

**Fig 1:**
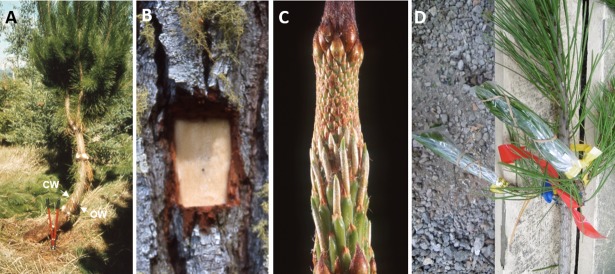
Tissues used to isolate RNA. A) compression (CW) and opposite wood (OW), B) developing xylem and phloem, C) developing buds and D) on-tree needles inoculated and un-inoculated with *Phytophthora pluvialis* spores.

All trees sampled were New Zealand Forest Research Institute research trees, with the exception of tree1. Tree 1 was identified as a malformed 6 year old tree within a commercial forest of Kaingaroa Timberlands LTD, who provided permission for us to sample the tree, as malformed trees are removed as part of routine thinning operations to improve the overall quality of a forest block. In order to generate a rich transcriptomic resource, that captured a wide subset of expressed genes and genetic variation, a range of *Pinus radiata* genotypes, tissue types were collected at different developmental and temporal stages (Table 1). To prevent degradation of RNA, each sample was harvested directly into liquid nitrogen and stored at -80°C. For the pilot genotype, Tree 1, developing xylem tissue were harvested from the bent stem (Fig 1A) of a 6-yr-old tree [35] by peeling away a bark window (Fig 1B) to expose the developing cambium [36]. Xylem was similarly collected for Trees 6–8, with phloem collected for Trees 6 and 7 by removing the underside of the bark window (Fig 1B). For Trees 2, 3, and 4, bud samples were harvested from growing vegetative meristems, preferentially during the early spring flush (Fig 1C), Tree 6 buds were collected in autumn. Needles were also harvested during the spring flush for Trees 2–5. For Trees 3 and 5, needles infected with a foliar pathogen, *Phytophthora pluvialis* (Pp), were also sampled. Inoculation of this material was performed by exposing individual branches of the trees to approximately 1x 10^4^ zoospores in a closed bag for 24 hours [N. Williams, pers. comm.] (Fig 1D). Fascicles with typical lesion development were collected at 7, 9 and 11 days post inoculation, and 5 cm of the proximal ends pooled into a single sample.

**Table 1:** Transcriptomes generated from the following tissues.

Genotype	Tissues	Date collected	Tree Owner	Tree location
Tree 1	6 year old Opposite wood xylem (OW)	Mar 2008	Waimangu Forest owned by Kaingaroa Timberlands	LAT -38.258 LON 176.414
	6 year old Compression wood xylem (CW)	Mar 2008		
Tree 2	Needles (N)	Nov 2012	Scion Clonal archive	LAT -38.156 LON 176.270
	Spring Buds (SB)	Nov 2012		
Tree 3	Needles (N)	Nov 2012	Scion Clonal archive	LAT -38.156 LON 176.270
	Needles (infected) (NI)	Nov 2012		
	Spring Buds (SB)	Nov 2012		
Tree 4	Needles (N)	Nov 2012	Scion Clonal archive	LAT -38.156 LON 176.270
	Spring Buds (SB)	Nov 2012		
Tree 5	Needles (N)	Nov 2012	Scion Clonal archive	LAT -38.156 LON 176.270
	Needles (infected) (NI)	Nov 2012		
Tree 6	Spring xylem (SPX) 1.4 metres	Nov 2000	Scion research Trial RO 664/3 Forest owned by Kaingaroa Timberlands	LAT -38.622 LON 176.345
	Summer xylem (SUX) 1.4 metres	Mar 2001		
	Autumn Buds (AB)	Mar 2001		
	Summer phloem (Ph) 1.4 metres	Mar 2001		
Tree 7	2 year old Seedling xylem (X)	Oct 2012	Scion Field Trial	LAT -38.155 LON 176.268
	2 year old Seedling phloem (Ph)	Oct 2012		
Tree 8	Summer xylem (SUX) 1.4 metres	Mar 2001	Scion research Trial RO 664/3 Forest owned by Kaingaroa Timberlands	LAT -38.622 LON 176.345

### RNA extraction

Total RNA was extracted from approximately 0.5–1.5 g of tissue using a modified CTAB method [36], and stored at -80°C in 10mM Tris-HCl buffer (pH 8.0). Approximately 25 μg of total RNA from each sample was treated with DNase I enzyme to remove contaminating genomic DNA (gDNA) prior to confirmation of RNA integrity and gDNA removal by agarose gel electrophoresis. Absence of contaminants was confirmed spectrophotometrically using a NanoDrop 1000 spectrophotometer (Thermo Scientific, Waltham, USA) and sample concentrations estimated using a Qubit RNA BR kit on the Qubit v1 fluorometer (Thermo Scientific).

### RNA sequencing

New Zealand Genomics LTD (NZGL, Dunedin, New Zealand) performed sequencing on the RNA extracted from all trees (Trees 1–8). RNA quality (18S and 26S ratios) and RNA concentration were determined using the RNA 6000 LabChip in an Agilent 2100 Bioanalyzer (Agilent Technologies, Santa Clara, California, USA). Thereafter, samples progressed through sequencing on the next-generation Illumina Hiseq 2000 sequencing platform (Illumina Inc. San Diego, California, USA). The total number of reads and RNA input metrics are summarised in S1 Table.

### Sequence assembly

All programmes and software used in this analysis are listed in Table 2. All raw read outputs from the Illumina Hiseq 2000 were filtered to remove reads containing only the 3' adaptor fragment. The remaining 'clean' short reads progressed to downstream sequence assembly programmes on a genotype-by-genotype basis, for alignment into contigs, scaffolds and finally unigenes. All nucleotides in raw reads from BGI were supplied with a Solexa technology-based quality score; conversions to Sanger quality scores, where required, were performed using MAQ (Mapping and Assembly with Quality) [37]. Two assembly programmes were used in this study: pilot assemblies with Tree 1 were performed using both SOAPdenovo [37] and Trinity v r2012-01-25 [38], while subsequent assemblies (Trees 2–8) were performed using only Trinity v r2013-01-25 [38]. Datasets in the Trinity assembly were treated as paired end. A final multi-genotype assembly (Trees 1–8, all tissues) was performed with Trinity v r2013-02-25 [38].

**Table 2:** Software tools used for short read sequence alignment and SNP detection.

Software	Function	Version	Reference
SOAPdenovo	*de novo* assembly		Li *et al*. (2008)
SOAPdenovo-Trans	*de novo* assembly	1.03	Xie *et al*. (2014)
Trinity	*de novo* assembly	r2012-01-25 r2013-01-25	Grabherr *et al*. (2011)
Trinity RNASeq	*de novo* assembly	r2013-02-25	Grabherr *et al*. (2011)
Velvet	*de novo* assembly	1.2.10	Zerbino et al. (2008)
Oases	*de novo* assembly	0.2.08	Schulz *et al*. (2012)
BWA	Global alignment	0.5.9-r16	Li and Durbin (2009)
Bowtie2	Global alignment	2.1.0	Langmead *et al*. (2012)
MAQ	Quality score conversion, global alignment, polymorphic site identification	0.7.1	Li, Ruan and Durbin (2008)
rtg-GA	Global alignment, polymorphic site identification	2.2.1	www.realtimegenomics.com
Mosaik	Global alignment	1.1.0021	Lee (2010)
GATK	Local realignment, polymorphic site identification	1.0.5777	McKenna *et al*. (2010)
BLAST	Similarity searching Basic Local Alignment Search Tool	2.2.28+	Altschul *et al*. (2012)
PERL	Scripting language for file manipulation	5.10.1	Christians *et al*. (2012)
SAMtools	Polymorphic site identification	0.1.14 0.1.19	Li *et al*. (2009)
Freebayes	Polymorphic site identification	0.6.5	https://github.com/ekg/freebayes

### SNP discovery workflows

To evaluate a different variant calling workflows, different software for the various steps were tested in a range of combinations (Table 3). For the Tree 1 SOAPdenovo assembly, three different combinations of global alignment, local alignment and SNP discovery tools were trialled (pipelines 1, 6 and 9 in Table 3). For the pilot genotype Tree 1 Trinity-based assembly, all 9 pipelines (Table 3) were performed. All software used default parameters unless otherwise described. For the individual assemblies of Trees 2–8, three different SNP discovery pipelines were applied: rtg-GA, SAMtools [39], and Genome Analysis Toolkit (GATK) [40]. All the scripts utilised to create different combinations of software supplied as S1 File.

**Table 3:** Different workflows applied for short read sequence alignment and SNP detection in Tree 1 pilot assemblies.

Pipeline	Quality score	Global alignment software	Local realignment software	Polymorphic identification
1	Solexa	BWA	-	SAMtools
2	Sanger	BWA	-	SAMtools
3	Sanger	BWA	GATK	SAMtools
4	Sanger	BWA	GATK	GATk
5	Sanger	BWA	GATK	Freebayes
6	Sanger	MAQ	-	MAQ
7	Solexa	rtg-GA	-	rtg-GA
8	Solexa	Mosaik	GATK	GATK
9	Solexa	Mosaik	GATK	freebayes

SAMtools (Sequence Alignment/Map) was used to reformat the outputs and identify sequence variants. All defaults were used, with the following exceptions: 1) to reduce the mapping quality for reads with a high number of mismatches, the coefficient to reduce the mapping quality of reads that had a higher number of mismatches was set to 50 as recommended for BWA alignments, 2) Bayesian inference was used to call variants using maximum-likelihood inference for the priors, 3) genotypes were called at the variant site, and 4) for areas of high coverage, (e.g., repeat regions), variants with a read depth greater than 100 were removed, as there is a known problem assembling abundantly expressed genes [32].

Default parameters were also used for GATK, however, some file reformatting was required. For BWA alignment files, SAMtools was first used to merge, sort and convert outputs into binary sequence alignment/map format (BAM) prior to performing local realignments in GATK. Picard (https://broadinstitute.github.io/picard/) was user to reorder files prior to running the GATK variant calling tool.

MAQ has a utility to convert Solexa quality scores to Sanger quality scores, a requirement for MAQ alignments, it also altered the BWA alignment. All defaults were used, with the exception that paired ends not mapping correctly were discarded.

From the original pilot study with the Tree 1 assemblies, rtg-GA, SAMtools [39], and Genome Analysis Toolkit (GATK) [40] variant calling algorithms were selected for both performance, and ease of use. Each algorithm was used independently to identify SNPs within each genotype. SNPs detected by multiple algorithms were only counted once when generating the total number of SNPs.

SNP markers that were homozygous within an individual were not detectable using the genotype-by-genotype approach (e.g. A/A in one genotype and T/T in another genotype). Therefore, to identify SNPs that were variable between genotypes, the raw reads from each genotype were mapped to the multi-genotype transcriptome using Bowtie2 version 2.1.0 [41], and polymorphisms identified using SAMtools [39].

### Prediction of exons

Neves et al 2013 [42] reported that the presence of undocumented introns within target regions impacted the efficiency of sequence capture in their exome-capture genotype-by-sequencing platform. To predict intron/exon boundaries within the multi-genotype transcriptome, the assembly was aligned to the *Pinus taeda* ver 1.01e genome [43] using the Trinity assembly pipeline, as it was reported that the latest version (at that time) of Trinity outperformed SOAPdenovo with respect to % gene recovery [44].

### Filtering SNPs

After SNP discovery via the mapping of raw reads back to the assemblies, additional filtering was performed to increase the likelihood of detecting biologically real SNPs and not sequencing or alignment errors. Firstly, SNPs had to be located more than 10 bp from the edge of contig. Secondly, read depth at SNP locations had to be greater than or equal to 10. Thirdly, as each genotype was from a diploid individual, heterozygous SNPs within an individual should display approximate allele frequencies of 0.5 across all reads; we widened our criteria to allow minor allele frequencies of no less than 0.25 within an individual.

## Results

### Transcriptome assembly in individual genotypes

A total of 18 transcriptome libraries were sequenced (S1 Table), and assembled (Table 4), from a total of 1.75 billion reads. The number of contigs generated per genotype ranged from 112,461 to 240,053. The N50 contig size ranged from 19,320 to 35,503 bases per genotype. The raw data files are available at NCBI (www.ncbi.nlm.nih.gov/bioproject/482145).

**Table 4:** Summary of transcriptome assemblies for each genotype using Trinity v2.0.

Tree ID	Tissues^1^	Total trimmed Contigs	Total length bases (b)	Min contig (b)	Median contig (b)	Mean contig (b)	Max contig (b)	N50 Contig^2^	N50 Length (b)^3^	N90 Contig^4^	N90 Length (b)^5^
Tree 1^6^	OW, CW	240,053	189,954,978	201	384	791	16,502	35,503	1,517	160,376	288
Tree 1^7^	OW, CW	137,228		201			9,175				
Tree 2	N, SB	174,382	135,676,827	201	377	778	11,558	26,833	1,504	116,553	281
Tree 3	N, SB	144,891	128,260,169	201	417	885	13,455	22,746	1,735	92,347	309
Tree 4	SB, N, NI	164,911	140,803,864	201	419	853	11,048	26,095	1,625	107,140	305
Tree 5	N, NI	194,849	142,994,312	201	350	733	11,536	28,358	1,433	132,473	267
Tree 6	SPX, SUX, AB, Ph	223,427	189,323,752	201	420	847	16,579	34,701	1,591	145,615	727
Tree 7	SUX, Ph	122,659	114,034,559	201	505	929	9,798	21,562	1,672	78,394	346
Tree 8	SUX	112,461	110,811,316	201	511	985	12,357	19,320	1,819	70,137	359

^1^ See Table 1 for tissue codes

^2^ N50 contig is the number of large contigs that collectively contain 50% of the nucleotide bases.

^3^ N50 Length is the length of the shortest N50 contig.

^4^ N90 contig is the number of large contigs that collectively contain 90% of the nucleotide bases.

^5^ N90 Length is the length of the shortest N90 contig.

^6^ SOAPdenovo assembly

^7^ Trinity assembly

### Assembly of multi-genotype transcriptome

The 8 individual genotypes, previously assembled independently, were combined into a large “multi-genotype radiata transcriptome” containing 194,299 contigs, ranging in size from 201 bp to 16,575 bp, with an N50 of 1,434. When mapped to the *Pinus taeda* ver 1.01e genome assembly, 144,007 (74%) of the radiata contigs aligned to the *P*. *taeda* genome. This enabled the prediction of 449,951 putative exons, corresponding to 46,342 *P*. *taeda* scaffolds.

### Variant calling in the pilot genotype (Tree 1)

#### SOAPdenovo assembly

Three different combinations of global and/or local alignment, followed by variant calling (pipelines 1, 6 and 9 in Table 3), were tested on the Tree 1 SOAP*denovo* assembly. However, many of the raw ‘clean’ reads could not be remapped onto the unigene sequence, suggesting a problem with this approach. The best result was achieved with Mosaik (combination 9), although only 36% of the raw reads remapped to the unigene sequence. This unexpectedly low rate of realignment meant that a high number of polymorphisms were likely missed. Therefore, the decision was made to perform a new assembly using Trinity, and a combined transcriptomic library contig set was created for Tree 1, comprising 137,228 different contigs sequences, ranging in length from 201 to 9,175 bp.

#### Trinity assembly

Variant calling was undertaken using all nine different combinations of global alignment, and/or local alignment, and SNP identification software packages (Table 2) for the Tree 1 Trinity assembly. In all cases, the percentage of raw ‘clean’ reads that remapped was much higher than with the SOAP*denovo* assembly, ranging from 82% (rtg-GA) to 93% (BWA and Mosaik). For this reason, no further analyses were performed using the SOAP*denovo* assembly, and all subsequent reporting of SNPs were identified solely from the Trinity assemblies. SNPs were regarded as high confidence when all of the following criteria were satisfied: 1) more than 10 bases from the edge of the unigene, 2) allele frequency between 0.25 and 0.75, 3) a read depth of 10 or more sequences at that SNP position, and 4) at least 60 bases clear of other polymorphic features on at least one side of the SNP.

The number of high confidence SNPs (Table 5) ranged from 8,061 (pipeline 1) to 86,815 (pipeline 7), with 34,996 being the average number of SNPs detected across all combinations. Merely the conversion of sequence quality scores from Solexa to Sanger caused an additional 24,127 SNPs to be detected (pipeline 1 vs 2), while the addition of a subsequent local realignment step did not markedly change the SNPs detected (pipeline 2 vs 3). Changing the final polymorphic identification software from SAMtools to either GATK or Freebayes reduced SNP numbers by 9,232 or 19,445, respectively (pipeline 3 vs 4 or 5). Use of two other independent packages, MAQ (pipeline 6) and rtg-GA (pipeline 7), both without a local realignment step, gave the highest SNP predictions at 63,488 and 86,815, respectively. Pipelines 8 and 9 both used the original Solexa sequence quality scores and a Mosaik global alignment followed by a GATK local realignment, but differed in the SNP prediction software used. Approximately half as many SNPs were predicted using Freebayes in pipeline 9 (17,138) than for GATK in pipeline 8 (37,575).

**Table 5:** Pair-wise analysis of SNPs predicted among pairs of pipelines. Diagonal line represents SNPs unique to that combination, with the number of total quality SNPs identified by each method shown in the final row.

	Pipeline 1	Pipeline 2	Pipeline 3	Pipeline 4	Pipeline 5	Pipeline 6	Pipeline 7	Pipeline 8	Pipeline 9
Pipeline 1	2,251	4,175	4,161	800	1,080	2,730	3,873	758	782
Pipeline 2		46	32,048	4,670	7,623	15,470	21,184	4,117	4,677
Pipeline 3			28	4,651	7,615	15,450	21,159	4,116	4,671
Pipeline 4				1,663	1,385	6,106	6,764	7,808	863
Pipeline 5					6,598	8,111	11,506	1,301	2,643
Pipeline 6						8,684	41,058	6,325	7,153
Pipeline 7							16,194	7,246	7,897
Pipeline 8								21,846	2,060
Pipeline 9									31,154
Total quality SNPs identified	**8,061**	**32,188**	**32,124**	**22,892**	**14,679**	**63,488**	**86,815**	**37,575**	**17,138**
Quality scores	Solexa	Sanger	Sanger	Sanger	Sanger	Sanger	Solexa	Solexa	Solexa
Global alignment software	BWA	BWA	BWA	BWA	BWA	MAQ	rtg-GA	Mosaik	Mosaik
Local alignment software	-	-	GATK	GATK	GATK	-	-	GATK	GATK
Polymorphism identification	SAMtools	SAMtools	SAMtools	GATK	Freebayes	MAQ	rtg-GA	GATK	Freebayes

SNPs predicted multiple times using different software package combinations were expected have a higher likelihood of being a true polymorphic event, therefore, we aimed to distinguish SNPs common to multiple pipelines from those predicted in only one pipeline. Using a pair-wise combination approach, each pipeline was compared to the others (Table 5). Pipelines 6, 7, 8 and 9 each predicted greater than 7,000 unique SNPs, i.e. SNPs not shared with any other combination.

A total of 164,145 different SNPs were predicted across all pipelines, with only six SNPs predicted in all 9 software combinations (Table 6). There were 10,905 SNPs detected in 5 or more combinations, only 6.6% of the total SNPs predicted. A total of 37,814 (23%) SNPs are predicted by at least 3 or more pipelines. A total of 88,464 SNPs (53.9%) were identified by only a single software pipeline. Such SNPs should be considered with less confidence for downstream applications, however, without further testing, we cannot rule out the fact that these predicted SNPs may actually be true polymorphisms.

**Table 6:** Frequency of SNP detection across all 9 discovery pipelines.

Number of pipelines detecting the same SNP	Number of SNPs
9	6
8	74
7	626
6	2,991
5	7,208
4	11,021
3	15,888
2	37,867
1	88,464
Total SNPs	164,145

### SNP discovery in individual genotypes

For SNP discovery within each of the genotypes, we selected the three best SNP prediction tools identified in the pilot study: rtg-GA, GATK and SAMtools [39]. Using individual Trinity v 2.0 [38] assemblies for each genotype, with Bowtie2-mapped raw reads [41], three pools of SNPs were generated per genotype using each of the three SNP prediction tools. These pools were screened using the same quality criteria as described for Tree 1 to identify high confidence SNPs. A cumulative total of 683,135 unique SNPs were identified across all pipelines in the 8 genotypes analysed (Table 7). The rtg-GA software predicted the greatest number of SNPs, followed by SAMtools then GATK. As SNP calling was performed within individuals, some redundancies are to be expected within this cumulative total; SNP discovery across genotypes will alter this total as SNPs are identified in multiple genotypes (decreasing the total number) and new SNPs are detected that were homozygous SNPs within genotypes (increasing the total number).

**Table 7:** Summary of SNP discovery within individual genotypes.

Genotype	Tissues^1^	SNP discovery algorithms			Total SNPs^2^	Unique SNPs^3^
		rtg-GA	GATK	SAMtools		
Tree 1	OW, CW	59,744	27,627	65,554	152,925	108,319
Tree 2	N, SB	58,320	23,192	53,715	135,227	92,232
Tree 3	N, SB	48,786	20,587	44,897	114,270	76,912
Tree 4	SB, N, NI	57,550	21,303	41,184	120,037	87,291
Tree 5	N, NI	63,650	15,023	39,853	118,526	89,516
Tree 6	SPX, SUX, AB, Ph	63,716	33,965	58,707	156,388	107,290
Tree 7	X, Ph	39,171	19,300	37,053	95,524	65,695
Tree 8	SUX	35,761	14,433	33,938	84,132	55,880
**Average**		**53,337**	**21,929**	**46,863**	**122,129**	**85,392**
**Cumulative total**		**429,698**	**175,430**	**374,901**	**977,029**	**683,135**^4^

^1^See Table 1 for tissue codes

^2^Total SNPs is the cumulative total for a genotype across the three algorithms.

^3^ All SNPs is the cumulative total for a genotype, with redundant detections across multiple algorithms removed.

^4^ Cumulative total for all genotypes; SNPS which may be counted multiple times if they appear in multiple genotypes.

### SNP discovery in multi-genotype transcriptome

For the final round of SNP discovery within the multi-genotype Trinity v 2.0 [38] assembly, raw reads were mapped back to the contigs using Bowtie2 [41] and SAMtools [39] used to predict polymorphisms, and use of rtg-GA discontinued. Filtering criteria were applied to remove SNPs that were identified in sequences with a read depth of less than 10 and/or or less than 10bp from the edge of a contig. A total of 328,981 unique SNP markers were identified within the multi-genotype assembly, including 59,424 between-genotype SNPs which were only identified when multiple genotypes were compared.

## Discussion

SNPs identified from tissue-specific transcriptomes can be an ideal resource [45] for candidate gene SNP discovery or genome-wide SNP identification using platforms such as Illumina’s Infinium [46] or Affymetrix’s Axiom (www.affymetrix.com) [20, 33]. During the development and evaluation of the various SNP discovery pipelines described in this work, genotyping platforms that utilise genotype-by-sequencing (GBS) of reduced representation genomic DNA, either through restriction enzyme digestion [47], or targeted exome capture [42] became more widely available. These methods capture and sequence all SNP markers within target region, rather than focussing on specific SNPs as with fixed array SNP chips. Therefore, the accuracy of *in silico* SNP predictions became less critical with these alternative genotyping platforms as preselected SNP markers were not required. However, the prediction of high confidence SNPs, described here, did influence the selection of genomic sites to target for an exome capture GBS assay.

### Sequence assembly

We tested a number of sequence assembly software packages, including Trinity, SOAPdenovo, and Velvet/Oases [48, 49] (data not shown), although the latter required more RAM than was available to us at the time. The unexpectedly low rate of realignment in the SOAPdenovo assembly (only 36% of the raw reads remapped to the unigene sequence) meant that a high number of polymorphisms were likely missed. This has been reported for other assemblers as well as SOAPdenovo when mapping back on assembled contigs [32]. The Trinity package therefore outperformed during the remapping of the raw reads back against the assembled contigs, despite generating 43% fewer contigs than the SOAPdenovo assembly with a smaller maximum contig length (9,175 vs 16,502). Remapping was an essential component of SNP discovery, therefore the best performing assembly package for this step was selected ahead of the usual quality metrics of contig number, length or N50.

### Variant calling

The variation in SNP discovery observed in a single dataset, channelled through a range of bioinformatic pipelines, varied by over an order of magnitude, (8,061 SNPs with pipeline 1, up to 86,815 SNPs for pipeline 7), and highlights the extent of variation among SNP calling pipelines, with 54% of the 164,145 SNPs discovered in the Tree 1 pilot study being unique to a single pipeline. A similar study comparing SNP discovery pipelines in Antarctic fur seals [33] showed that, between the 4 different methods compared, only 51% of SNP markers were detected in more than one pipeline. The filtering criteria applied post variant calling is also an important consideration, as low representation of an allele within a total read depth could be a dubious variant or sequencing error [19]. However, using these tools in combination can provide a more robust pipeline for SNP discovery, and we are starting to see this approach of applying multiple variant calling tools to a sequencing dataset being adopted [20, 50]. A single variant tool can still be suitable depending on the downstream application, or where added confidence can be gained through other approaches, such as stringent mapping of segregating markers in full-sibling populations [51–53].

There are two processes that current NGS algorithms can employ in the discovery of polymorphic loci: (a) global and local sequence alignment, and (b) polymorphism detection. The Burrows-Wheeler alignment (BWA) can be used to assemble short sequence reads [54] then SAMTools can be applied for polymorphism discovery [39]. The Genome Analysis Toolkit (GATk) can be used for quality control, local and global sequence assembly [40], as well as for polymorphism discovery [55]. A third program, MOSAIK, is another open-source global assembly tool [56] which can be used in conjunction with FreeBayes to use Bayesian methods for detection of polymorphisms within short read alignments [57].

## Conclusion

The purpose of this work was to evaluate bioinformatic workflows and combinations of software for identification of polymorphic loci and the development of a resource for a number of genomic tools for the radiata pine industry, with a requirement for varying SNP densities. A large, complex genome and incomplete reference resources precluded whole genome resequencing for SNP marker discovery in radiata pine. Nine different pipelines applied to a single pilot transcriptome identified SNPs at a rate the ranged over an order of magnitude. However, the utilisation of transcriptomic RNA sequencing in combination with several variant calling pipelines and quality filtering, has identified 683,135 *in silico* SNP markers and 449,951 exome templates, the first large-scale SNP resource reported for this species. In addition, the *P*. *radiata* multi-genotype transcriptome assembly is proving to be a valuable resource and being utilised in multiple downstream projects, including facilitating the assembly of a *P*. *radiata* reference genome, various gene discovery programmes, pedigree reconstruction and genomic selection.

## Supporting information

S1 Table RNA sequencing summary. (DOCX)

S1 File Individual scripts used to perform analysis in this study. (TGZ)
