# Supplementary material for: Approaches to variant discovery for conifer transcriptome sequencing
Source: PLoS One. 2018 Nov 5;13(11):e0205835. doi: 10.1371/journal.pone.0205835 (PMC6218030; doi:10.1371/journal.pone.0205835)
Supplement: S1 Table — (DOCX) [file pone.0205835.s001.docx]

**S1 Table. RNA sequencing summary**

| **Pilot genotype** | **Tissue^1^** | **RNA concentration (Agilent 2100) (ng/ul)** | **RIN^2^** | **Megabases of sequence** | **Total number of reads** | **%>Q20** |
| --- | --- | --- | --- | --- | --- | --- |
| Tree 1 | CW | 2180.0 | 7.6 | 4,844 | 53,822,222 | 97.31 |
| Tree 1 | OW | 1686.0 | 6.8 | 4,744 | 52,711,114 | 97.34 |
| **Genotype** |  | **RNA concentration (Qubit) (ng/ul)** | **RIN^2^** | **Megabases of sequence** | **Total number of reads** | **%>Q30^3^** |
| Tree 2 | N | 263 | 4.3 | 13,042 | 129,134,552 | 89.62 |
| Tree 2 | SB | 216 | 7.3 | 8,040 | 79,609,278 | 89.80 |
| Tree 3 | N | 228 | 6.7 | 9,770 | 96,722,528 | 89.41 |
| Tree 3 | SB | 204 | 8.5 | 7,247 | 71,742,168 | 89.58 |
| Tree 4 | N | 60 | 5.7 | 10,026 | 99,267,160 | 89.54 |
| Tree 4 | NI | 318 | 5.9 | 9,253 | 91,620,374 | 89.58 |
| Tree 4 | SB | 206 | 4.3 | 10,916 | 108,077,304 | 90.18 |
| Tree 5 | N | 285 | 6.0 | 12,016 | 118,976,862 | 89.48 |
| Tree 5 | NI | 221 | 6.1 | 8,849 | 87,613,580 | 89.58 |
| Tree 6 | AB | 246 | 8.0 | 13,686 | 135,503,666 | 91.06 |
| Tree 6 | P | 299 | 8.9 | 9,994 | 98,952,754 | 90.77 |
| Tree 6 | SPX | 532 | 7.7 | 8,922 | 88,338,102 | 91.18 |
| Tree 6 | SUX | 331 | 6.6 | 15,962 | 158,035,568 | 91.06 |
| Tree 7 | P | 252 | 7.6 | 7,279 | 72,071,022 | 90.83 |
| Tree 7 | X | 420 | 5.2 | 10,637 | 105,317,404 | 91.09 |
| Tree 8 | X | 580 | 7.9 | 10,898 | 107,903,110 | 90.75 |
|  |  |  |  |  |  |  |
| ***TOTAL*** |  |  |  | ***166,537*** | ***1,648,885,432*** |  |

**^1^See Table 1 for tissue codes**

**^2^RNA Integrity Number**
